# The rapid spreading of monkeypox virus is a threat for global public health: What should we do to fight this old enemy?

**DOI:** 10.1002/hsr2.876

**Published:** 2022-10-04

**Authors:** Mst. Sarmin Akter, Md. Sohan, Md. Rabiul Islam

**Affiliations:** ^1^ Department of Pharmacy University of Asia Pacific Dhaka Bangladesh

**Keywords:** disease outbreaks, monkeypox, monkeypox virus, Poxviridae, public health, variola virus

## 1 BACKGROUND

A zoonosis known as monkeypox (MPX) is brought on by the monkeypox virus (MPXV), a kind of Orthopoxvirus similar to the smallpox virus, a member of the Poxviridae family.^1^, ^2^ The MPXV was initially identified in 1958 at a laboratory in Copenhagen, Denmark, had an epidemic of a pox‐like illness among monkeys. A newborn baby aged 9 months was the first human MPX case identified in 1970 in the Democratic Republic of the Congo (DRC). Then, six MPX confirmed cases were identified until May 1971 in West African nations. Since then, the virus has been verified in thousands of humans across 15 different nations.^3^ The first epidemic beyond the African nations was seen in the United States in 2003 after the importation of infected animals from Ghana.^4^ Since MPX re‐emerged in Nigeria in 2017, a few individuals affected with MPXV have been reported outside Africa among people who have recently traveled to Nigeria or contacted people visiting Nigeria.^5^ In 2018, three MPX cases were detected in the United Kingdom, Israel, and Singapore for the first time, demonstrating that the virus was exported from Nigeria to countries other than its endemic regions. In 2021, two people affected by MPXV were detected in the United States among travelers from Nigeria, one in Maryland and the other in Texas. In the central, northwestern, and southwestern parts of Africa, 25 suspected individuals were identified on February 17, 2022, along with three verified cases and two fatalities. On March 14, 2022, six MPXV cases with two fatalities were reported from the Central African Republic. From the first day of 2022 to mid‐April 2022, throughout 14 territories of DRC, about 1,152 MPX probable cases were recorded in 54 health centers, including 55 fatalities with a mortality rate of 4.8%. From the first day of 2022 to April 30, 2022, Nigeria reported 46 probable cases of MPX, including 15 confirmed cases.^6^ The European region recorded the highest confirmed cases of MPX. Two types of MPXV circulate in endemic areas—the Central African (Congo Basin) clade and the West African clade; the latter causes less severe disease and mortality.^7^

## 2 THE GLOBAL PUBLIC HEALTH THREAT

MPX was first discovered in Africa, but now it has spread worldwide in different countries. By the end of July 2022, more than 22,000 cases have been reported in 80 countries worldwide, almost all outside West and Central Africa, where the virus was endemic.^8^, ^9^ In the last week of July 2022, the World Health Organization (WHO) declared MPX as a Global Public Health Emergency.^10^ Previously, the WHO declared a global health emergency regarding six health‐related events.^11^ COVID‐19 was the most recent before the current MPX outbreak. The transition of COVID‐19 from epidemic and pandemic was very swift, causing significant harm to society, the economy along with physical and mental health.^12^, ^13^, ^14^ Therefore, the rapid spreading of MPXV and the declaration of health emergency by the WHO have created panic worldwide.

A concern is that death outside Africa was reported on July 29, 2022, for the first time. The total death due to MPXV is eight in the current outbreak, with five and three deaths reported in and outside Africa, respectively. In Brazil, the respected health ministry announced the first death due to MPX, who was a 41 years old man and a patient having lymphoma with an impaired immune system.^15^ So far in Brazil, about 1,066 people have been affected by MPX, and 513 probable virus cases have been reported. According to data published by the Ministry of Health, Brazil, the men who have sex with men (MSM) are prone to be affected by MPXV as till now, 98% of MPX cases are identified as MSM. Shortly after that, the Spanish Ministry of Health identified the first death in Europe from the virus, a patient suffering from encephalitis. Spanish officials verified a second fatality connected to MPX on July 30, 2022. According to the health ministry, roughly 120 of the 3,750 MPX cases required hospitalization.^16^ Therefore, individuals who have never visited Africa or are unrelated to African animals in endemic regions have also contracted MPX. Moreover, recent studies suggest that the potential mutation of pathogenic Orthopoxvirus species is higher than thought before.^17^ This phenomenon has alarmed scientists worldwide; a new threat to the global healthcare system is knocking on the door.

Moreover, infectious disease outbreaks affect the mental health and well‐being of the population with their physical health impacts. We noticed that the COVID‐19 pandemic has tremendously impacted the mental health of people across the world.^18^, ^19^, ^20^, ^21^ Mental health‐related problems significantly increased after the COVID‐19 pandemic due to health safety measures, social issues, economic crises, educational or career interruptions, and so forth.^22^, ^23^, ^24^, ^25^ During the COVID‐19 pandemic, the new rival MPX outbreak has created panic worldwide.^17^ Similar to the COVID‐19 pandemic, the MPX outbreak will further increase psychological problems worldwide as both infectious diseases have similar health safety guidelines. Fear of getting infected and death play an important role in people's attitudes and behavior related to infectious disease outbreaks.^26^, ^27^ Therefore, we recommend awareness about MPXV, care for mental health, social interaction, and the application of terror management theory to reduce additional mental health burdens during the ongoing MPX outbreak.

## 3 MPX INFECTION AND STIGMA

The recent outbreaks of the MPX virus in nonendemic countries have been shaped in the public eye by the stigma that MPX has been detected in dense sexual networks of MSM.^28^ As of June 7, 2022, the UK Health Security Agency (UKHSA) reported 302 confirmed MPX cases in England, the majority of them being young homosexual or bisexual males and MSM.^29^ Among the MPX cases, 99% of individuals are men in the United Kingdom.^30^ However, global health authorities do not classify MPX as a sexually transmitted infection. It is too quick to declare that the MPXV solely affects the MSM. Therefore, according to available epidemiological data, the lesbian, gay, bisexual, transgender, queer, and intersex (LGBTQI) community can be considered a group at a high risk of MPX infection.^31^ The disproportionately high rate of MPX cases in the LGBTQI population might worsen the stigma against that group. However, pathogens have no innate ability to discriminate based on sexual orientation, religion, nationality, race, or ethnic group. Therefore, people from all regions can be infected by this virus.^32^ “Stigma and discrimination can be as dangerous as any virus,” said WHO Director‐General Tedros Adhanom Ghebreyesus.^33^ The UNAIDS expresses concern about stigmatized behavior against the LGBTQI community and people in Africa. The WHO employs public institutions and the media to adopt a strategy for avoiding stigma regarding MPX.^31^

## 4 TRANSMISSION, SYMPTOMS, AND DISEASE SEVERITY

Direct contact with bodily fluids of an infected individual, mucous membranes, damaged skin, open rashes, or contact with materials that have been exposed to the virus are all ways MPXV can be spread. In addition, the virus can be transmitted between people through direct and prolonged face‐to‐face contact through droplet transmission.^34^, ^35^ Typically, MPX requires 6–13 days for incubation, although it can take between 5 and 21 days. The illness usually lasts 2–4 weeks. The illness usually begins with fever, headache, muscle aches, and fatigue.^35^ A rash appears at the initial infection site within 3 days and quickly spreads all over the body. The majority of human MPX cases experience mild to moderate symptoms. Inflammation of the brain, dehydration, inflammation of the cornea, conjunctivitis, and pneumonia are among the complications in the affected individuals. Mortality from MPX ranges up to 11% in outbreaks in endemic areas, and mortality primarily affects young children.^6^

## 5 THERAPEUTIC AND PREVENTIVE OPTIONS

As a countermeasure for smallpox and MPX infections, three Orthopoxvirus inhibitors have been used. Cidofovir, Brincidofovir (CMX001), and Tecovirimat (ST‐246) are the available authorized poxvirus inhibitors. All these three antiviral drugs showed good efficacy against Orthopoxvirus in animal models. However, their effectiveness in immune‐compromised subjects is limited since severe infections occur due to Orthopoxvirus in immune‐deficient humans and animals.^3^

Studies demonstrate that the vaccine used for smallpox immunization offers protection against other Orthopoxvirus species, including the MPXV.^36^ Eighty‐five percent protection against MPXV was found in individuals vaccinated against smallpox.^3^ The vaccinia virus vaccine (ACAM2000) and the Aventis Pasteur smallpox vaccine are the second generation of a replication‐competent smallpox vaccine. In 2003, ACAM2000, recommended by the Centers for Disease Control and Prevention (CDC) for MPXV endemic in the United States, was shown to relieve symptoms but failed to prevent the disease.^36^

## 6 PUBLIC HEALTH MEASURES

Public education about possible risk factors and MPX prevention strategies should be a priority for global health officials. The WHO has warned about an increase in MPX cases arriving from different countries as a result of the continuous social spreading of MPXV. Animals in laboratories or in the wild should not be handled without protection. Fully cooked foods derived from meat and animal body parts need to be consumed to lower the danger of virus transmission from the animal. To stop the virus from spreading, more limitations must be put on the international commerce of animals. The feasibility and appropriateness of antiviral drugs against the smallpox vaccine and MPX should be validated through clinical trials. Countries should develop prioritized immunization strategies for their most vulnerable citizens. Additionally, the MSM community should be vaccinated against preventable sexually transmitted diseases, use condoms, avoid unprotected sex, and have a healthy sex life.^17^ Additionally, to address the new MPXV outbreak, authorities should put the lessons discovered from the COVID‐19 outbreak into practice. These lessons relate to public education, prevention, treatment, and mental health care.^37^ We have learned from AIDS that blaming or stigmatizing a particular community can quickly undermine the response to an outbreak. Therefore, health officials should take the lead in promoting information to lessen the stigma associated with MPX transmission and the involvement of a particular group. They should connect communities with religious groups, community leaders, and health professionals to prevent the risk of communication of MPX infection. Health officials can message communities that most MPX cases are identified among gays or bisexuals, but the infection is rarely confined to a particular community or geographic boundary. More significantly, to minimize stigma and bigotry, the WHO should rename “monkeypox” as soon as feasible.^31^, ^38^

## 7 CONCLUSION

MPX has become a new threat to global health because of its rapid spreading. It is crucial to assess and plan as soon as possible since the shockingly significant proportion of MPX cases seems to portray a health disaster. It is high time to propose control techniques to counter the ever‐increasing danger. However, MPX disease is mild, and mortality is low. We should not panic about the recent outbreak of this infection. Stigmatizing language, behavior, or attitudes toward specific groups can undermine the response to the outbreak. To stop this outbreak from becoming a pandemic, contact tracing, prompt screening and quarantine of individuals, adequate measures, public education, and appropriate treatment are all advised.

## AUTHOR CONTRIBUTIONS

Mst. Sarmin Akter and Md. Sohan devised the study and wrote the first draft. Md. Rabiul Islam edited and revised the manuscript. Both authors reviewed and approved the final submission.

## CONFLICT OF INTEREST

The authors declare no conflict of interest.

## TRANSPARENCY STATEMENT

The lead author Md. Rabiul Islam affirms that this manuscript is an honest, accurate, and transparent account of the study being reported; that no important aspects of the study have been omitted; and that any discrepancies from the study as planned (and, if relevant, registered) have been explained.

## Data Availability

Data sharing is not applicable to this article as no new data were created or analyzed in this study.
